# Race Strategies of Open Water Swimmers in the 5-km, 10-km, and 25-km Races of the 2017 FINA World Swimming Championships

**DOI:** 10.3389/fpsyg.2019.00654

**Published:** 2019-03-21

**Authors:** Santiago Veiga, Luis Rodriguez, Pablo González-Frutos, Archit Navandar

**Affiliations:** ^1^Department of Health and Human Performance, Technical University of Madrid, Madrid, Spain; ^2^Royal Spanish Swimming Federation, Madrid, Spain; ^3^Catalonian Swimming Federation, Barcelona, Spain; ^4^University Francisco de Vitoria, Madrid, Spain; ^5^Faculty of Sport Sciences, Universidad Europea de Madrid, Madrid, Spain

**Keywords:** pacing, tactics, competition, time-gap, end spurt

## Abstract

Despite literature on the pacing strategies of endurance sports, there is an existing lack of knowledge about the swimmers’ tactical decisions in the open water races. The aims of the present research were (1) to compare the pacing profiles and tactical strategies of successful elite open water swimmers (men and women) in the 5-km, 10-km, and 25-km races and (2) to relate these pacing strategies to the end race results. Intermediate split times, positions and gaps with leaders of the first ten swimmers classified in the 2017 FINA World Swimming Championships races were collected from the public domain and were related to the finishing positions. Overall swimming velocities of the 5-km races were faster than the 10-km (δ 0.03 ± 0.03 m/s) and the 25-km (δ 0.14 ± 0.01 m/s) events with male swimmers achieving relatively faster mean velocities than females in the 5-km (δ 0.12 ± 0.01 m/s) compared to the 25-km (δ 0.08 ± 0.01 m/s) events. Medallist swimmers achieved moderate faster overall velocities than finalists in the 25-km races (0.01 ± 0.01 m/s) only. Inter-level differences were detected in selected splits for each race distance. Pacing profiles presented lap to lap velocity improvements in the 5-km and men’s 10-km races (from +0.02 ± 0.00 to +0.11 ± 0.01 m/s) but also mid-race decreases in the women’s 10-km and on the 25-km races. Successful swimmers were located in the leading positions of the 5-km races but at mid-group in the first part of the 10-km and 25-km races, with time gaps with leaders of 15–20 s. Faster lap swimming velocities, mid-race leading positions and shorter time-gaps were only related to the finishing positions in the last lap of the 10-km and in the three last laps of the 25-km events, but also in the first lap of the women’s 5-km race. Despite different mid-race positioning, successful open water swimmers typically presented negative pacing profiles, a consistent control of mid-race gaps with leaders (15–20 s maximum) and great spurts (4–6% faster than mean race velocities) at the end of races. Coaches and swimmers should be aware of the different race dynamics depending to the event distance in order to select optimal race strategies.

## Introduction

The Federation Internationale de Natation (FINA) first included the 5-km, 10-km, and 25-km open water events in the 2000 edition of the World Swimming Championships held in Hawaii (United States). Since then, a great proportion of the open water elite competitors have taken part in two or three of these distances within the same competition, as races are usually separated a minimum of 48 or 72 h from each other. For example, in the 2015 World Championships held in Kazan (Russia), 19 out of 24 male swimming participants in the 25-km event had previously taken part in the 5-km or 10-km race of that edition^1^. Despite these data, there are few examples of competitors who achieved a successful position in two open water events within the same competition. If data from the 2015 edition are taken into consideration, only one out of nine male medallists was able to repeat the podium position (in the 5-km and 25-km). This could indicate that the race strategies at this elite standard differed according to the race distance and duration. The same pacing distribution or tactical positioning favorable for a successful performance on the 5-km or 10-km event might not necessarily be the most suitable for the 25-km event.

The pacing distribution in endurance sports has been a well-explored topic in the literature so far and it is widely acknowledged that the adoption of an optimal energy distribution in efforts with controlled conditions will have a clear impact on performance by delaying the onset of fatigue (Foster et al., 2005). From an individual athlete perspective, the optimal energy output will depend on the ability to understand their own metabolic capacities in relation to the task demand (Roelands et al., 2013), which may be assisted by prior experience (Micklewright et al., 2009) to anticipate the end-point of the exercise (Highton et al., 2017). Mainly, three general pacing strategies have been described for the duration of the effort (positive, negative or even pace profiles) depending on the athlete performing the first half of the task duration at a faster, slower or with no velocity variations than the second half. Depending on the task duration as well as the athlete competitive level, the adoption of one strategy will be indicated, being the positive pacing profile recommended for short-duration (<1 min) (Tucker et al., 2006), the even profile for ultra-endurance (>1 h) (Tan et al., 2016) and the negative profile more observed in the middle-duration efforts (≈4 min) (Abbiss and Laursen, 2008).

Beyond these general strategies, some pacing profiles (U-shaped, J-shaped or reversed J-shaped) have been described in endurance events as parabolic profiles (Garland, 2005) according to the variations in velocity in specific parts of the race, like the first or the last ≈10% of the race distance. These variations usually respond to the tactical decisions employed in head-to-head competitions (Corbett et al., 2011) where, depending on extrinsic factors like the opponent’s behaviors (Smits et al., 2014) or the race configuration in packs (Hanley, 2015), competitors accelerate or decelerate to increase their chances of race success (Konings et al., 2016). Examples of these tactical decisions would be the fast speed naturally adopted by endurance competitors at the beginning of races before slowing down for most of the race distance (Tucker et al., 2006) or the velocity spurts observed at the end of races when athletes aim for the medal finishing positions (Thiel et al., 2012). Female athletes, in this aspect, have been reported to perform fewer pacing variations along races probably due to their physiological but also psychological specific features (Deaner et al., 2015).

In the open water swimming events, where the race duration ranges from 1 to 4 h, the tactical decisions of competitors seem to be of a critical importance because of the drag reduction when swimming behind or next to other competitors (Chatard and Wilson, 2003). Also, depending on the situation within the race, competitors interact in close contact with surrounding swimmers or can avoid collisions, which certainly influences their pacing decision-making throughout the race (Renfree et al., 2013). In 2017, Rodriguez and Veiga (2017) showed that successful competitors in the FINA World Swimming Championships 10-km event performed a conservative race strategy, with a delayed partial positioning and with swimming paces similar to those of non-successful swimmers for a great proportion of the race distance. With this strategy, they were able to save energy for an end spurt that was highly related to race success. However, it is unknown whether this same strategy would be effective for the remaining open water distances (5-km and 25-km). Despite the great increase in participants and races in the last years, there is a lack of information about the optimal race strategies for each open water event (Baldassarre et al., 2017).

Therefore, the aims of the present research were (1) to compare the pacing profiles and tactical strategies of successful elite open water swimmers (men and women) in the 5-km, 10-km, and 25-km races and (2) to relate these pacing strategies to the end race results. It was expected that swimming paces would be faster in the shorter races, with a more conservative strategy of successful swimmers (both in the swimming pace and the tactical positioning) in the longer events (25-km) compared to the shorter events (10-km and 5-km). Also, it was expected that female swimmers would demonstrate lower pacing variations within and between the race distances.

## Materials and Methods

Data from the 5-km, 10-km, and 25-km races of the FINA World Swimming Championships held in Budapest in July 2017 were freely available on the website (see footnote 1). The three races were held in a 7-day period with race circuits structured, as usual, in laps of 2.5 km: in this way, competitors had to perform 2, 4 or 10 laps, respectively, for the 5-km, 10-km, and 25-km events, to cover the total race distances. The FINA Organising Committee confirmed the official distances of each race according to the global positioning system coordinates of the official reference marks situated along the race circuits. All experimental procedures were carried out in accordance with the Declaration of Helsinki and were approved by the Technical University of Madrid’s ethics committee.

From all the finishing competitors in the three distances (61, 65, and 25 swimmers for the men’s and 57, 59, and 19 for the women’s 5-km, 10-km, and 25-km, respectively), the first ten swimmers classified in each race (top-10) were selected for further analysis and were considered as successful participants. This criterion was made according to previous research (Rodriguez and Veiga, 2017) and to the fact that the Olympic classification in the open water disciplines corresponds to the top-10 swimmers in the World Championships event. In total, 30 males and 30 females race results (10 per each race distance) were included into the present research and were subsequently divided in medallists (swimmers classified first to third) or finalists (swimmers classified fourth to tenth).

The race strategies in the 5-km, 10-km, and 25-km events were defined by registering the split and end race times, the mid-race and finishing positions (from first to tenth) and the gap-times (s) with the leading swimmers in each of the 2.5 km laps of the race circuit. Mean swimming velocities (m/s) in each lap as well as at the end of the race were calculated from official split and end times. These velocities were expressed as time per 100 m (s) for better interpretation purposes. Performance density (%) of each race was expressed as the difference between the mean swimming velocity of the first and tenth classified swimmers related to the velocity of the race winner (Baldassarre et al., 2017). In order to facilitate comparison between race distances, mid-race parameters were expressed in relation to the percentage of the total race distance covered at that point. Also, mid-race positions were indicated relative to the total participants in each race (Figure 2).

Statistical analyses were performed using IBM SPSS statistics for Windows, version 20.0 (IBM Corp, Armonk, NY, United States). Swimming paces of successful swimmers were compared with a repeated measures analysis of variance according to the race distance (5-km, 10-km, and 25-km), the race lap (first to second in the 5-km, first to fourth in the 10-km and first to tenth in the 25-km race), gender (male or female) and competitive level (medalist or finalist). Planned repeated contrast tests between successive laps were carried out. *Post hoc* tests were used to determine statistical effects (*p* < 0.05) between factors using Bonferroni corrections and were interpreted using effect sizes (partial η^2^) with 0.2, 0.5, and 0.8 threshold values for small, medium and large effects (Cohen, 1992). The race tactical behaviors (mean velocity, mid-race positions and gap-times) were related to the end race results by using Pearson correlation coefficients, being 0.1, 0.3, 0.5, 0.7, and 0.9, the threshold values that represented small, moderate, large, very large, and nearly perfect correlations (Hopkins et al., 2009). Data variability was reported using standard deviations (SD) and intra-individual coefficient of variation (CV) and the uncertainty of estimates was indicated using 90% confidence intervals (CIs).

## Results

### Overall Trends According to the Race Distance, Sex, and Competitive Level

The mean swimming pace in the open water 2017 FINA World Swimming Championships races (Table 1) showed meaningful differences according to the race distance (*F*_2_ = 5043.75, *P* = 0.001, η^2^ = 0.99) being faster in the 5-km when compared to the 10-km (δ 0.03 m/s; 90% CI, 0.03–0.03 m/s, *P* = 0.001) and to the 25-km (δ 0.14 m/s; 0.13–0.14 m/s, *P* = 0.001) events. Male swimmers swam faster mean velocities than their female counterparts in all events (*F*_1_ = 7759.08, *P* = 0.001, η^2^ = 0.99) although relatively, gender differences in velocity were greater in the 5-km event (δ 0.12 m/s; 90% CI, 0.11–0.12 m/s, *P* = 0.001, η^2^ = 0.98) than in the 10-km (δ 0.10 m/s; 0.10–0.11 m/s, *P* = 0.001, η^2^ = 0.97) or the 25-km (δ 0.08 m/s; 0.08–0.09 m/s, *P* = 0.001; η^2^ = 0.96) races. Also, medallists swam faster mean velocities than finalists swimmers (*F*_1_ = 35.11, *P* = 0.001, η^2^ = 0.42) although meaningful differences were only observed in the 25-km event (0.01 ± 0.01 m/s, *P* = 0.001, η^2^ = 0.45). Performance density of the top-10 swimmers (indicating velocity differences between first and tenth classified swimmers) was 0.51% and 0.61% for the 5-km, 0.33% and 0.38% for the 10-km and 1.13% and 2.38% for the 25-km, respectively, men’s and women’s races.

**Table 1:** Swimming pace (per 100 m) of medallists and finalists’ participants on the 5-km, 10-km, and 25-km events of the 2017 FINA World Swimming Championships.

Males		Total	Split 1	Split 2	Split 3	Split 4	Split 5	Split 6	Split 7	Split 8	Split 9	Split 10
5-km	Medal	65.50 ± 0.12	67.38 ± 0.07	62.61 ± 0.19								
	Final	65.75 ± 0.02	67.64 ± 0.31	62.83 ± 0.31								
10-km	Medal	67.19 ± 0.00	69.48 ± 0.56	68.31 ± 0.26	67.52 ± 0.32^∗^	62.77 ± 0.19						
	Final	67.29 ± 0.08	69.04 ± 0.22	68.47 ± 0.10	67.83 ± 0.12	63.14 ± 0.28						
25-km	Medal	72.67 ± 0.01^∗^	75.80 ± 0.05	74.95 ± 0.14	74.91 ± 0.33	71.61 ± 0.36	75.34 ± 0.04	73.16 ± 0.06	72.43 ± 0.13	70.86 ± 0.17	71.37 ± 0.03	66.72 ± 0.09^∗^
	Final	73.07 ± 0.01	75.92 ± 0.33	74.81 ± 0.33	75.23 ± 0.39	71.31 ± 0.30	75.23 ± 0.29	73.33 ± 0.18	72.51 ± 0.31	71.18 ± 0.87	71.28 ± 1.15	70.23 ± 1.99
Females		Total	Split 1	Split 2	Split 3	Split 4	Split 5	Split 6	Split 7	Split 8	Split 9	Split 10
5-km	Medal	70.99 ± 0.05	72.97 ± 0.21	67.92 ± 0.18^∗^								
	Final	71.30 ± 0.12	73.17 ± 0.19	68.33 ± 0.19								
10-km	Medal	72.16 ± 0.02	75.16 ± 0.92	71.89 ± 0.64^∗^	72.73 ± 0.19	68.11 ± 0.36						
	Final	72.27 ± 0.08	74.86 ± 0.39	72.36 ± 0.30	72.78 ± 0.26	68.33 ± 0.36						
25-km	Medal	77.29 ± 0.02^∗^	81.81 ± 0.15	80.79 ± 0.22	78.97 ± 0.20	75.84 ± 0.60	83.76 ± 1.01	79.67 ± 0.23	72.29 ± 0.23	71.34 ± 0.07^∗^	75.32 ± 0.21^∗∗^	73.55 ± 0.21
	Final	78.37 ± 0.61	81.51 ± 0.24	80.64 ± 0.27	78.75 ± 0.36	76.25 ± 0.48	83.81 ± 0.62	80.00 ± 0.29	72.51 ± 0.44	75.50 ± 3.31	79.19 ± 1.86	75.82 ± 2.21

*Medallists are statistically faster (p < 0.05) than finalists with moderate^∗^ or large^∗∗^ differences.*

### Pacing Profiles of the Successful Competitors on the 5-km, 10-km, and 25-km Races

**FIGURE 1:**
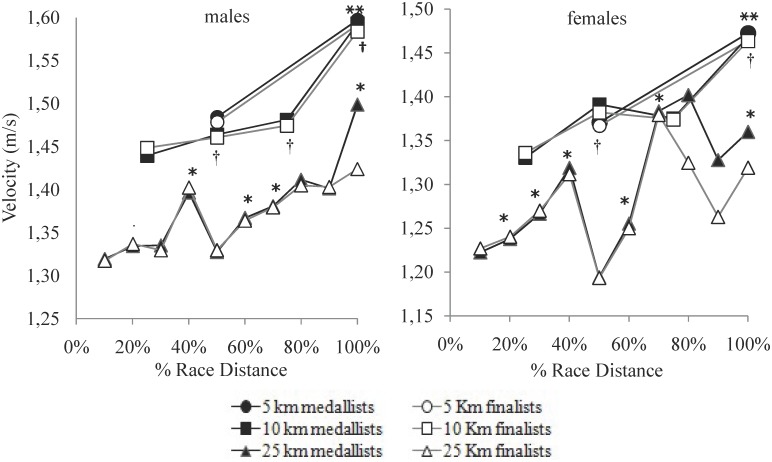
Swimming velocity (m/s) evolution of the medallists (black) and finalists (white) on the 5-km, 10-km, and 25-km events of the 2017 FINA World Swimming Championships (error bars removed for clarity). Swimming lap velocity is statistically faster (*p* < 0.05) than the previous 5-km (_^∗∗^_), 10-km (†) and 25-km (_^∗^_) lap for the mean top-10 swimmers.

The evolution of the swimming pace throughout the open water races (Figure 1) showed meaningful changes both in the 5-km (*F*_1_ = 1826.79, *P* = 0.001, η^2^ = 0.99), the 10-km (*F*_2.07_ = 1033.44, *P* = 0.001, η^2^ = 0.98) and the 25-km events (*F*_4.21_ = 139.92, *P* = 0.001, η^2^ = 0.89). These changes depended on the gender (lap gender: η^2^ = 0.32, 0.64 and 0.58, respectively, in the 5-km, 10-km, and 25-km events) although some small differences (lap level: η^2^ = 0.17 and 0.28 in the 10-km and 25-km races, respectively) were also observed between medallists and finalists.

In the 25-km event, the planned contrasts showed significant increases between successive laps (*p* < 0.001) except between the seventh and the eighth laps. *Post hoc* tests showed that the swimming velocity of male and female competitors showed large increases from lap to lap (at a maximum rate of +0.07 ± 0.01 m/s and +0.13 ± 0.01 m/s, for males and females) except in the fifth, eighth, and ninth laps. In these laps, the swimming pace was maintained (eighth lap) or even decreased (fifth lap: −0.07 ± 0.02 m/s and −0.12 ± 0.02 m/s, for males and females, both *P* = 0.001) (Figure 1). Finalists swam at similar paces than medallist swimmers except in the last lap (tenth lap differences = 0.08 m/s; 90% CI, 0.03–0.13 m/s, *P* = 0.01, η^2^ = 0.39) of the male’s race and on the eight (0.08 m/s; 0.02–0.13 m/s, *P* = 0.01, η^2^ = 0.37) and ninth (0.07 m/s; 0.03–0.10 m/s, *P* = 0.001, η^2^ = 0.51) laps of the females’ race.

In the 10-km event, the planned contrasts showed a significant increase from the first to the second lap (*p* < 0.001) and from the third to the fourth laps (*p* < 0.001). *Post hoc* tests showed that the swimming pace also increased from lap to lap on the men’s race (second lap: +0.02 ± 0.01 m/s, *P* = 0.01; third: +0.02 ± 0.00, *P* = 0.001 and fourth: +0.11 ± 0.01 m/s, *P* = 0.001) but it decreased on the third lap of the women’s race (−0.01 ± 0.01 m/s, *P* = 0.001) before the spurt of the fourth lap (+0.09 ± 0.01 m/s, *P* = 0.001). This was observed in all the successful swimmers of the 10-km event, except the men finalists’ group who did not increase swimming pace on the second lap (*P* = 0.23).

Finally, in the 5-km event, the planned contrasts showed a significant increases from the first to the second lap (*p* < 0.001) as well as the *post hoc* tests both for male (+0.11 ± 0.01 m/s, *P* = 0.001, η^2^ = 0.99) and female (+0.10 ± 0.01 m/s, *P* = 0.001, η^2^ = 0.98) swimmers, regardless of the competitive level. Medallists and finalists’ swimmers in the 5-km and 10-km races did not show inter-level velocity differences at any race lap, except in the second lap of the 5-km (0.01 m/s; 0.00–0.02 m/s, *P* = 0.05, η^2^ = 0.23) and 10-km (0.01 m/s; 90% CI, 0.00 to 0.02 m/s; *P* = 0.05, η^2^ = 0.22) women’s races and in the third lap on the 10-km men’s race (0.01 m/s; 0.00 to 0.01 m/s; *P* = 0.04, η^2^ = 0.24).

Changes in the swimming pace in relation to the mean race velocity reached maximum values in the last lap of races, with a magnitude of 6.71% and 5.81% (for male and female swimmers) in the 10-km event, 5.51% and 3.89% in the 25-km and 4.63% and 4.40% in the 5-km races. Intra-individual coefficient of variation in the lap velocity of the longest event (25-km) reached 6.48% and 9.65%, respectively, for the men’s and women’s race.

### Tactical Positioning of the Successful Competitors on the 5-km, 10-km, and 25-km Races

**FIGURE 2:**
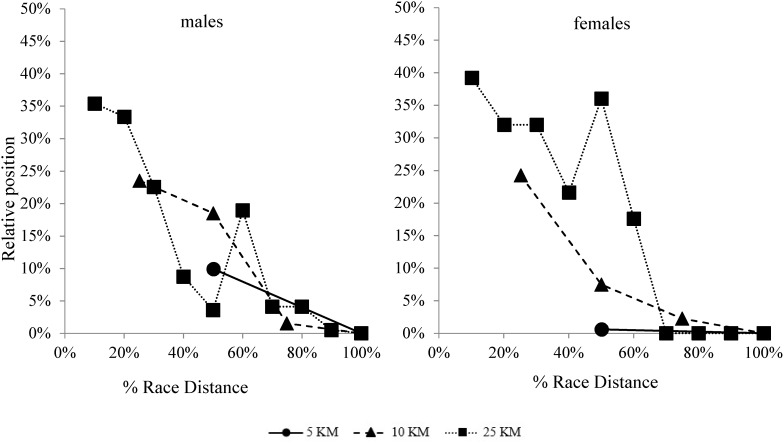
Mean partial positioning (relative to the total participants) of the successful participants (top-10) on the 5-km, 10-km, and 25-km events of the 2017 FINA World Swimming Championships (error bars removed for clarity).

**FIGURE 3:**
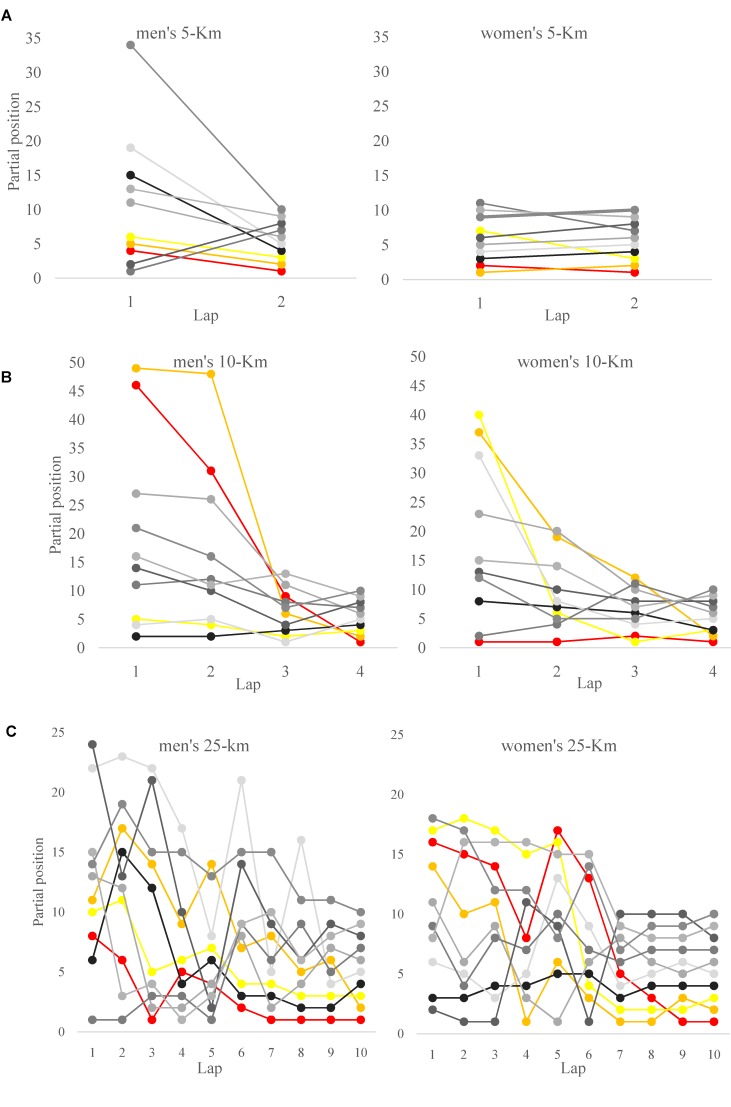
Lap-to-lap positioning of the successful participants (top-10) on the **(A)** 5-km, **(B)** 10-km, and **(C)** 25-km events of the 2017 FINA World Swimming Championships.

**FIGURE 4:**
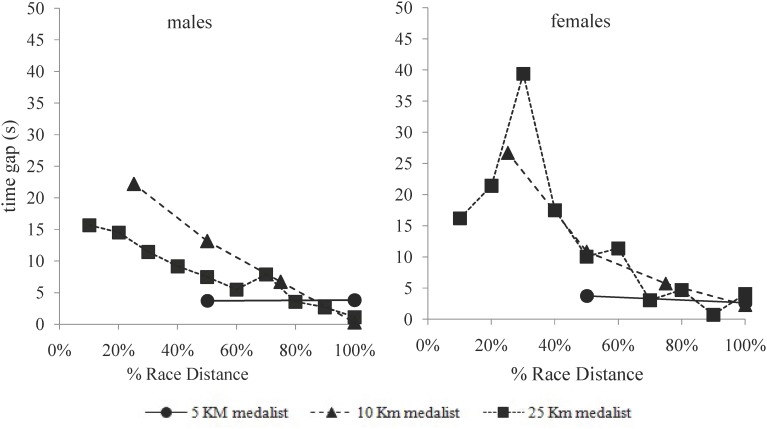
Mean time gap (s) with leaders of the successful swimmers (top-10) in the 5-km, 10-km, and 25-km events of the 2017 FINA World Swimming Championships (error bars removed for clarity).

In relation to the tactical positioning (Figure 2–4), successful swimmers of the 5-km events (males and females) were located at the 2.5-km split in the 20% front part of the group within a 10 s gap from the leading swimmers. Top-10 participants of the 10-km events, for their part, showed a more delayed positioning in the first half of the race (around the 40–50% front part of the main group) with a time gap of 15–20 s with the leaders. In the second half of the race, however, they reduced the time gap with leaders to 10–15 s and moved up to the 15% front part of the group. Finally, in the 25-km races, successful male swimmers showed a similar tactical positioning to the 10-km race swimmers by locating in the middle of the group during the first 60% of the race distance, 15–20 s beyond the leaders. However, in the second part of the race, their time gap with leaders decreased to less than 10 s and their partial positioning was within the top-10 swimmers. Successful female swimmers in this event, in turn, showed a more delayed positioning in the first half of the race by locating in the rear part of the group and more than 20 s behind leaders.

### Relationships of Mid-Race Parameters With End Race Result

The relationships between the mid-race parameters and the end race result of the successful swimmers in the 2017 FINA World Swimming Championships (Table 2) indicated that the swimming pace in the first three and six laps of the 10-km and 25-km events (the 70% and 60% of the race distance), respectively, was not statistically related to the swimmer performance. Indeed, the swimming lap velocities of the 25-km race were only related to the race result in the men’s last lap and in the women’s last three laps of the races. In the 5-km race, however, the swimming velocity of the female swimmers in each race lap (first and second) was related to a better final race position. For the mid-race positions and time gaps with leaders, these parameters were not related to the 5-km or 10-km end race results, except for the females 5-km event. Tactical positioning, however, showed meaningful correlations with the race success in the last three laps of the 25-km race distance, both for male and female swimmers.

**Table 2:** Relationships between lap swimming velocities, partial positions and intermediate gaps (from the first to the last lap) and the end race results for the top-10 competitors (males and females) of the 5-km, 10-km, and 25-km races of the 2017 FINA World Swimming Championships.

25-km		Lap 1	Lap 2	Lap 3	Lap 4	Lap 5	Lap 6	Lap 7	Lap 8	Lap 9	Lap 10
Velocity	Male	−0.19 (−0.68 to 0.39)	0.36 (−0.22 to 0.77)	−0.38 (−0.78 to 0.20)	0.27 (−0.31 to 0.73)	0.23 (−0.35 to 0.71)	−0.39 (−0.79 to 0.18)	−0.46 (−0.82 to 0.10)	0.11 (−0.47 to 0.63)	−0.33 (−0.76 to 0.25)	−0.97^∗∗∗^ (−0.99 to −0.91)
	Female	0.21 (−0.37 to 0.69)	−0.07 (−0.61 to 0.49)	0.10 (−0.47 to 0.63)	−0.46 (−0.82 to 0.10)	0.34 (−0.23 to 0.76)	−0.53 (−0.85 to −0.01)	−0.30 (−0.74 to 0.29)	−0.87^∗∗^ (−0.96 to −0.64)	−0.88^∗∗^ (−0.97 to −0.68)	−0.82^∗∗^ (−0.95 to −0.54)
Position	Male	0.34 (−0.24 to 0.76)	−0.06 (−0.60 to 0.50)	0.21 (−0.38 to 0.69)	0.12 (−0.46 to 0.64)	−0.17 (−0.66 to 0.42)	0.57 (0.07 to 0.87)	0.74^∗∗^ (.36 to 0.92)	0.49 (−0.06 to 0.83)	0.84^∗∗^ (0.57 to 0.96)	
	Female	−0.24 (−0.71 to 0.34)	−0.06 (−0.60 to −0.51)	−0.15 (−0.65 to 0.43)	0.46 (−0.09 to 0.82)	−0.18 (−0.67 to 0.41)	0.31 (−0.28 to 0.74)	0.77^∗∗^ (0.43 to 0.94)	0.93^∗∗∗^ (0.80 to 0.98)	0.94^∗∗∗^ (0.83 to 0.98)	
Gap	Male	0.20 (−0.39 to 0.68)	−0.10 (−0.62 to 0.47)	0.22 (−0.37 to 0.70)	0.09 (−0.48 to 0.62)	−0.20 (−0.9 to 0.38)	0.16 (−0.42 to 0.66)	0.64^∗^ (0.18 to 0.89)	0.11 (−0.47 to 0.63)	0.68^∗^ (0.26 to 0.91)	
	Female	−0.21 (−0.69 to 0.37)	−0.08 (−0.61 to 0.49)	−0.10 (−0.62 to 0.47)	0.22 (−0.37 to 0.70)	−0.36 (−0.77 to 0.22)	0.27 (−0.31 to 0.73)	0.80^∗∗^ (0.48 to 0.94)	0.86^∗∗^ (0.63 to 0.96)	0.93^∗∗∗^ (0.82 to 0.98)	

10-km		Lap 1	Lap 2	Lap 3	Lap 4						
Velocity	Male	0.35 (−0.23 to 0.77)	−0.25 (−0.71 to 0.34)	−0.62 (−0.88 to −0.15)	−0.83^∗∗^ (−0.95 to −0.56)						
	Female	0.14 (−0.44 to 0.65)	−0.32 (−0.75 to 0.26)	−0.11 (−0.63 to 0.46)	−0.63^∗^ (−0.89 to −0.16)						
Position	Male	−0.40 (−0.79 to 0.17)	−0.40 (−0.79 to 0.17)	0.31 (−0.28 to 0.74)							
	Female	−0.28 (−0.73 to 0.30)	0.07 (−0.50 to 0.60)	0.23 (−0.35 to 0.71)							
Gap	Male	−0.35 (−0.77 to 0.22)	−0.37 (−0.77 to 0.21)	0.29 (−0.29 to 0.74)							
	Female	−0.14 (−0.65 to 0.44)	0.22 (−0.36 to 0.70)	0.35 (−0.22 to 0.77)							

5-km		Lap 1	Lap 2								
Velocity	Male	−0.44 (−0.81 to 0.12)	−0.32 (−0.75 to 0.27)								
	Female	−0.74^∗∗^ (0.37 to 0.93)	−0.64^∗^ (−0.89 to −0.18)								
Position	Male	0.47 (−0.08 to 0.82)									
	Female	0.78^∗∗^ (0.45 to 0.94)									
Gap	Male	0.44 (−0.12 to 0.81)									
	Female	0.74^∗∗^ (0.37 to 0.93)									

*Magnitudes of correlations with P-values < 0.05: ^∗^large; ^∗∗^very large; ^∗∗∗^nearly perfect.*

## Discussion

The present research aimed to compare the pacing profiles and tactical strategies of the open water swimmers (men and women) competing at the 5-km, 10-km, and 25-km races of the FINA 2017 World Swimming Championships. Successful swimmers in the three events performed a negative pacing profile with increasing swimming velocities at the end of races. However, the dynamic of pacing profiles as well as the tactical positioning of the competitors depended on the race distance and on gender.

### Overall Trends According to the Race Distance, Sex, and Competitive Level

Mean swimming paces in the 5-km, 10-km, and 25-km races showed clear differences from race to race and represented different swimming intensities according to the lactate threshold velocities of elite freestylers (Pyne et al., 2001). A high level of competitiveness was demonstrated by the mean swimming velocity of the top-10 swimmers, similar to that of recent Olympic 10-km races (1.49 m/s for men and 1.39 m/s for women in the present research compared to 1.49 and 1.39 m/s in Beijing 2008, 1.51 and 1.41 m/s in London 2012 and 1.47 and 1.41 m/s in Rio 2016, respectively, for men and women) and faster than that reported in other international 10-km events (Vogt et al., 2013). The density of performances of the top-10 swimmers in Budapest was also greater than previous 5-km (1.83% for men and 1.95% for women), 10-km (1.5% and 2.3%) and 25-km (3.76% and 3.31%) official open water races (Vogt et al., 2013; Zingg et al., 2014). As expected, medallists and finalists’ swimmers showed marginal velocity differences for the entire races, highlighting the tactical importance of major championships where athletes compete for the best finishing position but not the best possible time (Thiel et al., 2012; Casado and Renfree, 2018; Hanley and Hettinga, 2018).

Gender differences in the swimming velocity close to 7% were greater than previously reported (Vogt et al., 2013; Baldassarre et al., 2017) in the 10-km Olympic Games races in Beijing (6.3%), London (6.6%), and Rio (3.7%). These data disagree with previous results from non-conventional open water races such as the Manhattan Island Marathon Swim (Knechtle et al., 2014) and the “Maratona del Golfo Capri-Napoli” (Rüst et al., 2014), where the gender gap had continuously decreased over years. However, as previously observed (Knechtle et al., 2014), female swimmers in the present research presented a relatively better performance in the longer distances (9.30% gap between the 5-km and 25-km races for them compared to the 10.95% of male swimmers) with lower inter-gender differences in the 25-km (6.20%) than in the 5-km (7.80%) race times. The body composition of female athletes, with a more favorable percentage and distribution of fat tissue (Mclean and Hinrichs, 1998), would provide them with more buoyancy and less drag to perform better in the longer distances (Baldassarre et al., 2017). In relation to the pacing variations, female swimmers did not show lower changes in velocity than men as could be expected due to their physical features but also to their lower tendency to take risks in a race context (Deaner et al., 2015; Hanley, 2016). However, their end race spurts were of a lower magnitude than their male counterparts in line with the lower speed variations reported in the literature (Abbiss et al., 2013; Renfree and St Clair Gibson, 2013).

### Pacing Profiles of the Successful Competitors on the 5-km, 10-km, and 25-km Races

In the 5-km event, successful competitors of the men’s and women’s races situated on the leading positions from the beginning of race and adopted a swimming pace faster (approximately 2 s per 100 m) than that of the 10-km race. From this fast but maintainable pace, medallist and finalists further increased their swimming pace by ≈7% in the second half of the race and maintained their leading positions to aim for the final top three positions. This strategy had been previously reported a successful approach by proficient Marathon runners who want to be in control of the race (Hanley, 2016), as it prevented less capable competitors to increase pacing in the second half of races.

10-km male and female swimmers, on the other hand, adopted slower paces for the first three race laps of the race and showed moderate pacing variations (first to third laps pace change lower than 3%). However, in the last lap of the race, they performed a dramatical end spurt (by increasing swimming velocity by 9.7% and 6.6%, respectively), which was largely related to their race success. This end race spurt was greater than that of 1.5–3% reported in the 2015 FINA World Swimming Championships (Rodriguez and Veiga, 2017) and it is a typical feature of highly trained endurance athletes with a great anaerobic reserve who speed up at the end of races to ensure they cross the finish line ahead of other competitors (Corbett et al., 2011; Renfree et al., 2013). Previous studies on endurance disciplines have reported end spurts of 1–2% for the successful competitors at the end of running marathons (Renfree and St Clair Gibson, 2013; Hanley, 2016) or 5–8% in shorter events like track running (Thiel et al., 2012). By maintaining a relative even pace profile for most of the race distance (in this case, 75% of the total race distance), successful swimmers in the 10-km race probably chose the best option to save glycogen reserves (Padilla et al., 2000) and this allowed them to subsequently perform an end spurt of a greater magnitude (de Koning et al., 2011).

Finally, in the 25-km races, competitors showed a more variable pacing profile than in the shorter events. Swimming paces in the first splits of the race were relatively slow (6–7 s slower per 100 m than the 10-km competitors) but then, progressively, they were increasing from lap to lap although not in a linear manner (Figure 1). In particular, the coefficient of variation between laps reached values between 6 and 9% that are considerably greater than those of Olympic track races (Thiel et al., 2012) or running Marathons (Renfree and St Clair Gibson, 2013). Probably, swimmers in this event (4-h long) organized their energy output according to external race conditionings and giving less weighting to their physiological status. This could respond to the so-called “herd behavior,” where athletes follow the behavior of surrounding opponents regardless of their rational decision making (Tan et al., 2016).

When comparing the pacing profiles of finalists and medallists open water swimmers, results indicated similar swimming velocities and race dynamics for the most of race distance between them, but a greater ability of medallists to increase swimming pace at the end of races (Figure 1). This had been previously observed in Olympic track races (Thiel et al., 2012) and it was especially highlighted in the 25-km event, where medallists swam faster velocities than finalists in the last lap of the men’s and in the eighth and ninth lap of the women’s race only (Table 1). Probably, medallists presented similar aerobic capacities than finalists’ swimmers at the World Championships level but a better ability to achieve swimming paces below 65 (men) or 70 (women) seconds per 100 m, which are typical paces at the end of elite open water races and indicate a greater anaerobic reserve for them (Corbett et al., 2011).

In general, the pacing strategies of successful swimmers in the 5-, 10-, and 25-km races showed clear differences with those of other endurance disciplines. Running competitors had been reported (1) to adopt initial paces considerably faster than the mean speed of the race (Tucker et al., 2006), (2) to present the lowest pacing decreases in the second half of races (March et al., 2011; Deaner et al., 2015), and (3) to perform an end spurt in the last ≈10% race distance (Thiel et al., 2012). However, elite open water swimmers in the present research (especially in the 10-km and 25-km races) did not adopt a fast pace approach at the beginning of the race but they performed the slowest pace in the first split (both men and women). Also, in all the 5-km, 10-km, and 25-km events, successful swimmers did not decrease pace in the second half of races, but they presented negative pacing profiles instead. Mean times of top-10 swimmers in the second part of races were approximately 2, 3 or 7 min shorter (for the 5-km, 10-km, and 25-km events) than in the first half. In relation to the end spurts, open water swimmers seemed to speed up velocity over a greater proportion of race distance compared to other endurance disciplines, although this information should be confirmed by a greater temporal resolution of intermediate splits (Thiel et al., 2012). All these characteristics of open water pacing profiles probably responded to pre-planned race strategies related to the drag resistance of swimming, where competitors situated behind or at the side of other leading competitors could save up to 20% the energy cost (Chatard and Wilson, 2003). Open water swimmers may deliberately seek to save energy in the initial stages of races (by swimming slower than an ideal pace) and then regulate pace according to the race configuration in packs to avoid gaps that would decrease the drafting effect.

### Tactical Positioning of the Successful Competitors in the 5-km, 10-km, and 25-km Races

When examining the tactical positioning of successful swimmers in the different race distances, the 5-km event showed a different profile from that of the 10-km and 25-km races. Swimmers in the shortest event situated in the leading part of the main group from the early stages of the race (Figure 3) and with a narrow gap from leaders (shorter than 5 s or approximately 5 m). This was especially evident of the women’s race where the finalists were located in the top 11 positions for the entire race, with mid-race positions and short times gaps being largely related to the end race result. The early leading strategy was probably less dependent on the opponent’s behavior and, according to the shorter duration of the 5-km race, highlighted the ability of open water swimmers to deliver the best possible energy output for the duration of the race (Foster et al., 2005).

In the longer events (10-km and 25-km), successful competitors adopted a more conservative positioning in the first half of the race by locating in the mid-part of the main group. The time-gap with leading swimmers, in these events, did not exceed 15–20 s which represented (according to the swimming velocities) a maximum distance of 30–35 m. This had been previously observed in the 10-km race of the 2015 World Swimming Championships (Rodriguez and Veiga, 2017), but it had not been ever reported with time gaps in order to fully understand race dynamics. From the half of the race, both 10-km and 25-km successful competitors of the present study improved their relative positions and decreased the time gaps from leaders but, interestingly, 25-km male swimmers progressed to a more advance situation in the main group (within 10 s of leaders and in the 5% front positions of the main group) which was largely related to their race success (Table 2). The successful 10-km swimmers, on their behalf, did not achieve the leading positions until the last lap of the race [as previously observed during the 2015 World Championships (Rodriguez and Veiga, 2017)] assisted by their greater end spurt compared to the 5-km or 25-km events. Differences in the tactical positioning between events probably depended also on the different performance densities of races, as the greatest density of the 10-km was contrary to the lowest density observed in the 25-km race.

Regardless of the event distance, it was noticeable the ability of medallists across all distance events to maintain time gaps with leaders no longer than 10–15 s in the second half of races. These time-gaps (which would represent distance-gaps no longer than 20 m) allowed medallist to be in control of the race as they were able to cut them down within the 25–30 min duration (2.5 km) of the last race lap. It was also noticeable the influence of the mid-race positioning of medallist’s swimmers (and especially of winners) in the race pacing variations. For example, a relative backward movement of medallists within the main group at the fifth lap of the 25-km race (losing from two to five positions, Figure 4) was accompanied, both in the men’s and women’s race, by a decrease in the swimming pace of the top-10 swimmers in that race lap (Figure 1). These race behaviors highlighted the influence of the extrinsic factors on the tactical decisions of elite open water swimmers (Renfree et al., 2013; Smits et al., 2014) and explained the greater pacing variations presented in the longest open water race (25-km), due to greater changes in the partial positioning. Other extrinsic factors that could also influence the race outcomes were specific aspects of the race venue like the structure of the course, currents, water temperature, …etc. (Abbiss and Laursen, 2008). These environmental constraints were probably beyond the conscious strategy of the open water swimmers, but they certainly also affected their pacing and tactical positioning.

### Practical Applications

These results of the World Championships races highlight differences in the pacing profiles and tactical positioning of open water swimming compared to other endurance and ultra-endurance disciplines. Coaches and swimmers should be aware of the different race dynamics according to the event duration and should focus on the development a greater anaerobic reserve besides their great aerobic capacity. If properly handled through races, energy savings during the initial and mid stage of races due to drafting (especially in the longer distances) should be translated into great end spurts to leave behind adversaries and to access to the successful race positions. This could only be achieved if accompanied by an adequate control of time gaps with leaders within the main field, that would allow elite performers to reach the leading positions in the last lap of races.

## Conclusion

Successful swimmers in the different events of the 2017 FINA World Swimming Championships (5, 10, and 25-km races) performed a negative pacing profile with an increase of their swimming velocity at the end of races. However, while doing so, they employed different tactical positioning strategies that depended on the race distance, their final positioning and gender. In the 5-km event, successful competitors presented an early quick pace with an advance mid-race positioning (20% part of the main group and 10 s maximum gap from leaders) that it was largely related to the women’s end race result. 10-km competitors, on the other hand, adopted a slower than ideal pace with small pacing variations and a delayed partial positioning for most of the race distance. This allowed them to perform a dramatic end race spurt (between 6 and 9% velocity increase) in the last quarter of the race distance related to a better end race result. Finally, 25-km competitors performed a more variable pacing profile related to the greater changes on the mid-race positioning, but an aggressive strategy on the second half of the race with leading positions that allowed them to achieve race success. Regardless of the event distance, medallists swimmers showed a greater ability to control gap times with leaders around 10–15 s (≈15–20 m) for most of the race distance and to employ their greater anaerobic reserve to increase swimming pace at the end of races. Female swimmers, for their part, presented a relatively better performance in the longer events with more advance partial positioning that their male counterparts but a lower end race spurt.

## Author Contributions

SV, LR, PG-F, and AN contributed to the conception and design of the study. LR, PG-F, and SV organized the database. AN and SV performed the statistical analysis. SV and LR wrote the first draft of the manuscript. All authors contributed to manuscript revision, read and approved the submitted version.

## Conflict of Interest Statement

The authors declare that the research was conducted in the absence of any commercial or financial relationships that could be construed as a potential conflict of interest.

## Footnotes

1 http://www.omegatiming.com
